# Pax2 and Pax8 cooperate in mouse inner ear morphogenesis and innervation

**DOI:** 10.1186/1471-213X-10-89

**Published:** 2010-08-20

**Authors:** Maxime Bouchard, Dominique de Caprona, Meinrad Busslinger, Pinxian Xu, Bernd Fritzsch

**Affiliations:** 1 Biochemistry Department, Goodman Cancer Centre, McGill University, Quebec, Canada; 2 Department of Biology, College of Liberal Arts and Sciences, 143 Biology Building, Iowa City, IA 52242-1324, USA; 3 Research Institute of Molecular Pathology, Vienna Biocenter, Vienna, Austria; 4 Department of Genetics and Genomic Sciences, Mount Sinai School of Medicine of New York University, New York, NY 10029, USA

## Abstract

**Background:**

*Pax2;5;8 *transcription factors play diverse roles in vertebrate and invertebrate organogenesis, including the development of the inner ear. Past research has suggested various cochlear defects and some vestibular defects in *Pax2 *null mice but the details of the cochlear defects and the interaction with other *Pax *family members in ear development remain unclear.

**Results:**

We show that *Pax2;8 *double null mice do not develop an ear past the otocyst stage and show little to no sensory as well as limited and transient neuronal development, thus indicating that these two family members are essential for overall ear morphogenesis and sustained neurosensory development. In support of functional redundancy between Pax proteins, *Pax2 *can be substituted by a *Pax5 *minigene, a gene normally not expressed in the embryonic mouse ear. There is no detectable morphological defect in *Pax8 *null mice suggesting that *Pax2 *expression can compensate for *Pax8*. Conversely, *Pax8 *cannot compensate for *Pax2 *leading to a cochlear phenotype not fully appreciated previously: Cochlear development is delayed until E15.5 when the cochlea extrudes as a large sack into the brain case. Immunocytochemistry and tracing from the brain show that a cochlear spiral ganglia form as a small addition to the inferior vestibular ganglion. However, the empty cochlear sack, devoid of any sensory epithelium development as indicated by the absence of Sox2 or MyoVII expression, nevertheless develop a dense innervation network of small neurons situated in the wall of the cochlear sack.

**Conclusions:**

Combined these data suggest that *Pax2 *is needed for organ of Corti formation and is directly or indirectly involved in the coordination of spiral ganglion formation which is partially disrupted in the *Pax2 *null ears. All three *Pax *genes can signal redundantly in the ear with their function being determined primarily by the spatio-temporal expression driven by the three distinct promoters of these genes.

## Background

*Pax2;5;8 *genes are vertebrate *Pax *orthologs that evolved out of an ancestral *Pax2;5;8;6 *gene of sponges [1] that became associated with ocelli and statocysts in coelenterates [2]. *Pax2;5;8 *became associated with ear development in vertebrates and sensilla development in flies among additional expressions domains in brain, kidney and other organs. In vertebrates, *Pax8 *is among the earliest genes unequivocally expressed in the developing otic placode of fish, frog and mice and appears to be later largely co-expressed with *Pax2 *in the mouse ear. Several papers have at least partially characterized the effects of *Pax2 *inactivation in the mouse ear [3-5]. The data agree that *Pax2 *function is essential for cochlear development in mice and human but vary in the degree of vestibular defects and in the degree of loss of sensory neurons. However, as the cochlea is a mammalian novelty[6], the expression of *Pax2 *in the ear of bony fish that have not evolved a cochlea [7] reflects a more ancient function of *Pax2 *in vertebrate ear development. Moreover, *Pax2 *reduction in zebrafish ear development results in hair cell defects that may be initiated at the level of the otic placode [2,8]. In this system, *Pax2 *and *Pax5 *seem to regulate ear development downstream of *Fgf3/8 *with a similar near complete loss of ear differentiation in knock-down experiments [8-10]. In contrast to *Pax2*, previous work on *Pax8 *null mice has shown a thyroid phenotype but no obvious ear defect was identified [11]. *Pax5 *is apparently not expressed in the ear of mouse embryos and no defects have been reported in *Pax5 *mutants [12] in contrast to zebrafish embryos [8]. In chicken, *Pax8 *is lost and *Pax2 *appears to be the only gene relevant for placode invagination [13], clearly indicating the importance of these *Pax *genes for ear induction and development.

Consistent with the idea of functional conservation, an allelic series of *Pax2 *and *Pax8 *mutation indeed showed a dose dependent effect indicative of compensation of either gene by the other in the kidney [14]. We present here data on *Pax2;8 *double null mice showing a comparable gene dosage effect in the ear. We also show that loss of both *Pax2 *and *Pax8 *results in disruption of sensory formation, whereas the evolutionary novelty of the vertebrate ear, the sensory neurons [15,16] form at least transiently. Later roles of Pax2 in cochlear and sensory neuron patterning are revealed by the analysis of *Pax2 *null and *Pax2*^*5ki/5ki *^mice, a mouse in which the *Pax2 *gene was replaced by a *Pax5 *minigene [17]. Combined, these data show that, as observed in the kidney, multiple steps of ear development are exquisitely sensitive to *Pax *gene dosage.

## Results

**Figure 1:**
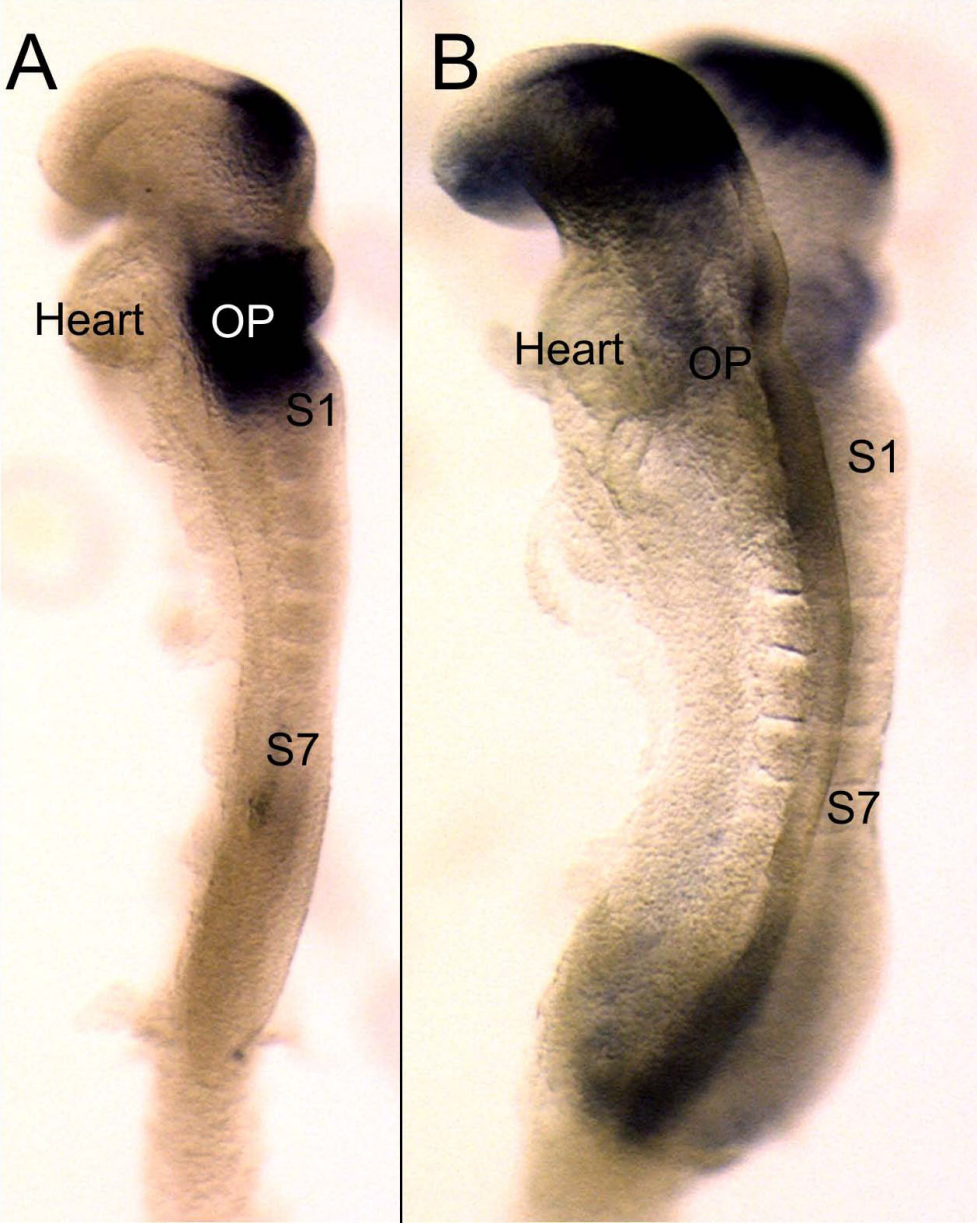
**Early expression of *Pax2 *and *Pax8 *compared**. This image shows the earliest expression of *Pax8 *(A) and *Pax2 *(B) in 7 somite (S1, S7) mouse embryos of approximately 8 embryonic days. Note that at this stage *Pax8 *is more profoundly expressed throughout the area of the future otic placode (OP) just anterior to the first somite (S1). In contrast, *Pax2 *is more prominently expressed in the brain with only very limited expression in the area of the otic placode (OP). At this stage the heart is anterior to the future otic placode. Bar indicates 100 um.

Previous work has shown that *Pax8 *is upregulated in zebrafish prior to *Pax2 *and a very early expression of *Pax8 *was also noted in mice in the otic region prior to somite formation but not directly compared with *Pax2 *expression in the same mouse lines. We verified that indeed this sequence of expression is also true for mice as previously suggested based on sections. Our data confirm that *Pax8 *is one of the earliest markers of the otic placode region that is prominently upregulated at the 7 somite stage (Fig. 1). In contrast, *Pax2 *is barely visible in the otic placode at this stage but has a much more profound expression in the brain. These data confirm and extend previous work and demonstrate that both *Pax2 *and *Pax8 *are expressed in early stages of placode formation in mice and zebrafish around the time mesenchyme specification induces the first postotic somites.

**Figure 2:**
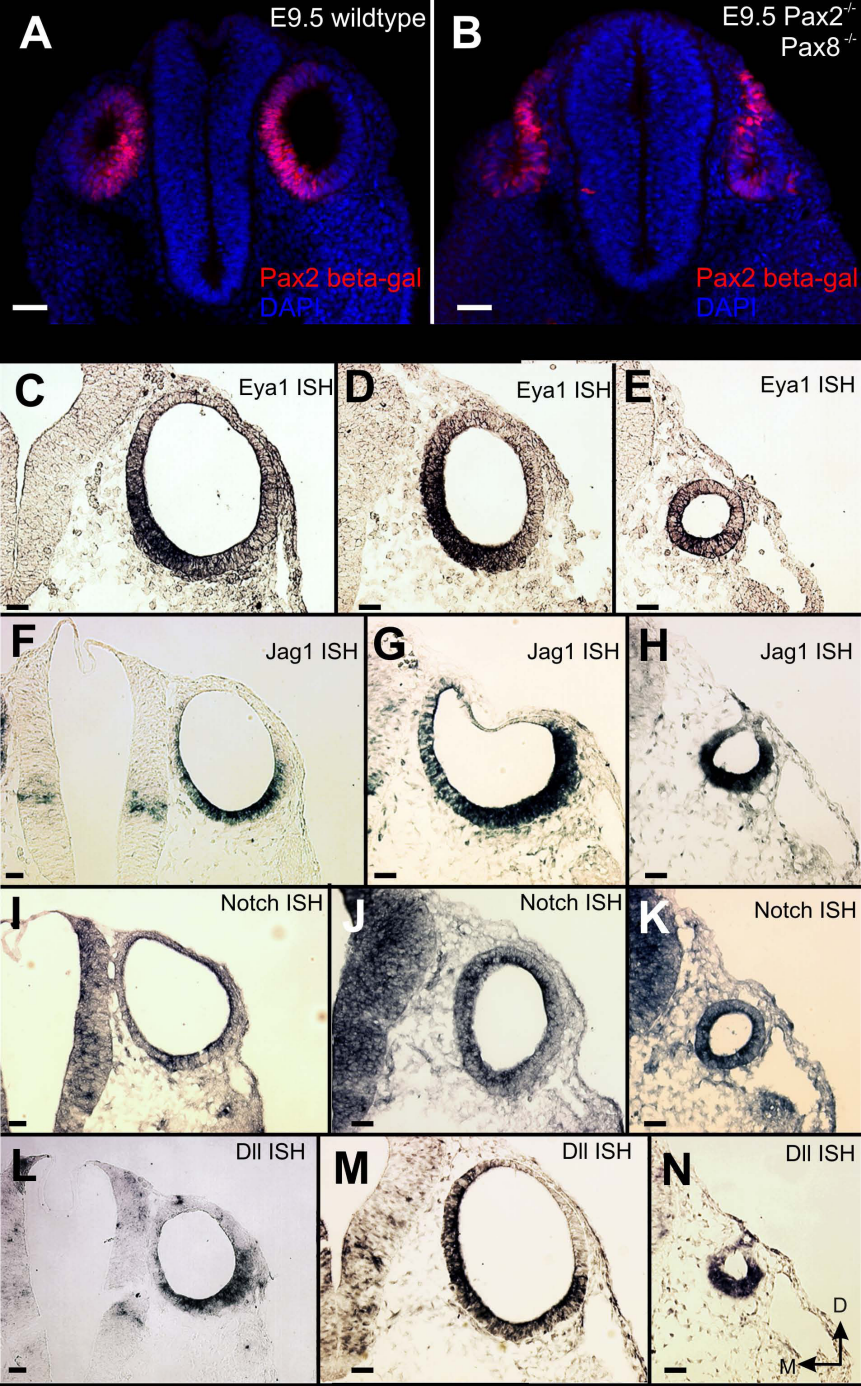
***Pax2;8 *affects invagination but not early marker expression**. Shortly after the otocyst is detached from the ectoderm, *Pax2 *expression is throughout the ventral and medial aspect of the developing ear (as revealed by a LacZ-reporter for *Pax2*). In contrast, the reporter for *Pax2 *shows in the *Pax2;8 *double null mouse a much wider expression including areas outside the partially invaginated otic placode. Expression profile of four genes known to be important for overall ear development (*Eya1, Dll*) and for neurosensory development (*Jag1, Notch*) show no expression changes in simple *Pax2 *or compound *Pax2;8 *double mutants, suggesting that *Pax2 *and *Pax8 *have little effect on these early markers. However, there is a reduction in otocyst size and a delayed detachment from the ectoderm in *Pax2;8 *double null mice. Bar indicates 100 um.

We next examined the further development of the otocyst in *Pax2;8 *double null mice and found that these mice have defects in the otic placode invagination at E9.5 as revealed by β-Galactosidase immunostaining expressed from the *Pax2 *targeted allele. At this stage, the vesicle has fully invaginated and detached from the ectoderm in wild-type embryos (Fig. 2A,B) but instead was defective in invagination and remained continuous with the ectoderm the *Pax2;8 *double mutant embryos. This suggests that the combined *Pax2;8 *double null affects otocyst morphogenesis (Fig. 2A,B), leading to a smaller otocyst, possibly through aberrant cell movements.

At E10.5 the overall size differences in the otocyst were more dramatic between *Pax2;Pax8 *double null and either *Pax2 *or wild type (Fig. 2C-N). Interestingly, none of the genes known to play a role in ear morphogeneis (*Eya1, Dll*) nor neurosensory development (*Jag1, Notch*) was lost in *Pax2;8 *double mutant ears. However, these markers revealed a somewhat ventralized fate of double mutant otocysts, in concordance with the loss of placode tissue due to incomplete invagination of the placode (see Fig. 2A,B). Combined these data suggest that the initial expression of *Pax2 *and *Pax8 *is supporting a process of otocyst invagination that fails to be completed in the absence of those two *Pax *genes whereas the expression of the tested genes for further sensory ear development is almost unaffected.

**Figure 3:**
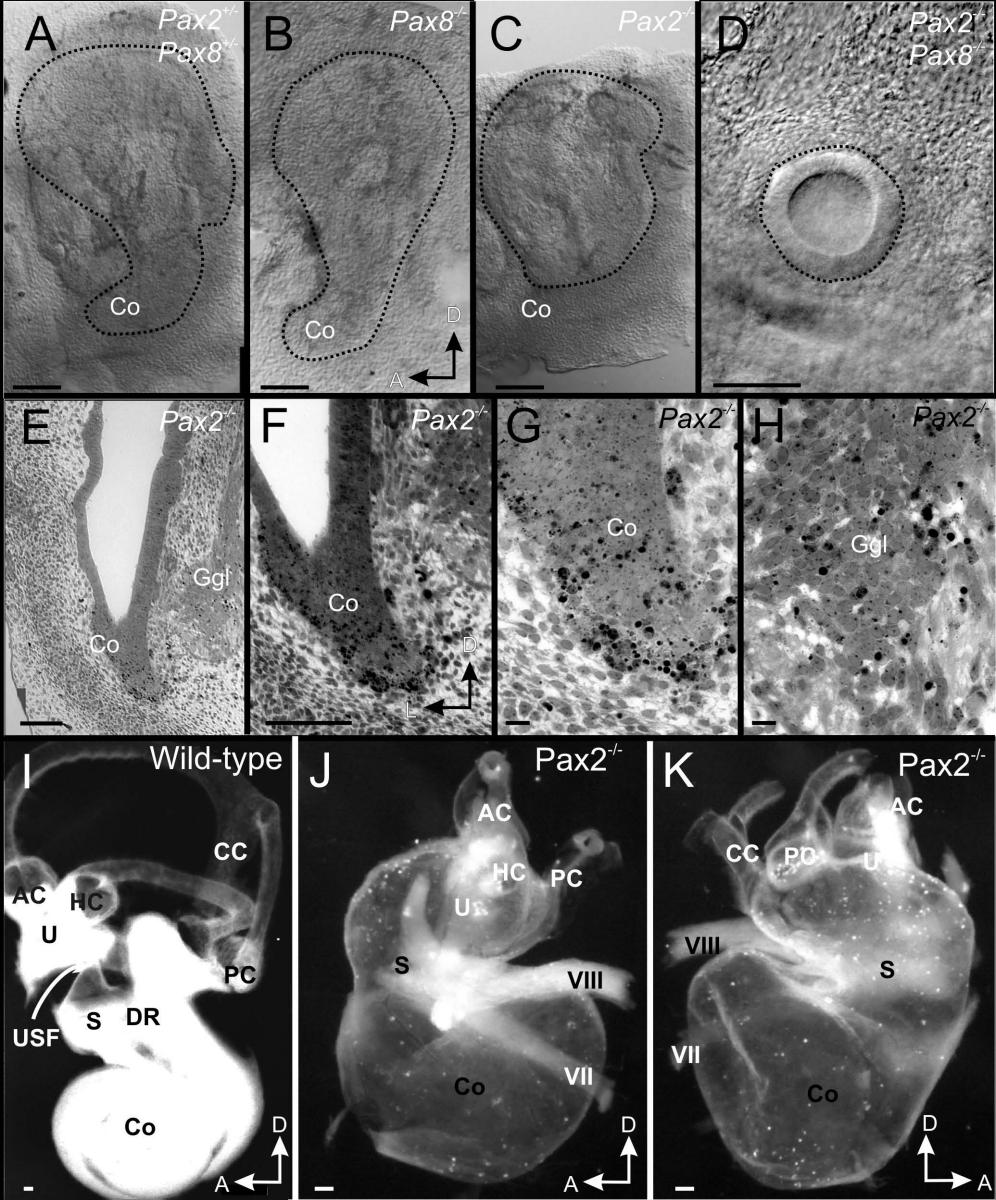
**Overview of ear defects and sections**. Whole mounted (A-D, I-K) and coronally sectioned ears (E-H) show the mutant effects on morphology. The cochlear duct is truncated in *Pax2 *null whereas *Pax2;8 *double null mice have only an otic vesicle at E11.5 (A, B; outlined by a dotted line). Coronal sections (see vertical line in C) show no cochlear outgrowth in *Pax2 *null mice as late as E13.5 (E). Numerous apoptotic cells in and around the cochlear duct (F, G) are indicated by chromatin condensation. Cartilage is obvious lateral but not medial to the ear (E). Higher magnification shows also apoptotic cells in the ganglion including in the more ventral, presumably spiral ganglion part (H). At E18.5 wild-type ears have developed a coiled cochlea, three distinct canals and recesses for the saccule and the utricle (I). The utricle is separated by the utriculo-saccular foramen from the saccule (USF in I) and the cochlea is separated from the saccule by the ductus reunions (DR in I). *Pax2 *null mice have an inflated saccule and cochlear duct with shortened semicircular canals (J, K). The only constriction resulting in two adjacent vesicles is near the saccule. Facial (VII) and vestibular (VIII) nerves are abutting the ear adjacent to the constriction separating the superior from the inferior division. AC, anterior canal/crista; CC, common crus; HC, horizontal canal/crista; Co, cochlear duct/sack; DR, ductus reunions; Ggl, ganglion; PC, posterior canal/crista; S, Saccule; U, utricle; USF, utriculo-saccular foramen; VII, facial nerve; VIII vestibular/cochlear nerve. Bar indicates 100 um in A-F, I-K, 10 um in G, L.

We next examined overall morphology of the developing ear using whole-mounts and sections. Consistent with previous work we found that the cochlear duct growth was reduced as early as E11.5 (Fig. 3) in *Pax2 *null mice. Neither *Pax8 *null mice nor combinations of *Pax8 *null with *Pax2 *heterozygosity showed such defects indicating that the cochlear defect is exclusive to the *Pax2 *function. This is consistent with absence of expression of *Pax8 *in the developing cochlea. However, combined deletion of *Pax2;Pax8 *resulted in small ear vesicles that were only a fraction of the size of wild type or *Pax2 *null ears (Fig. 3A-D).

Previous work had suggested that cochlear truncation in *Pax2 *mutant embryos comes about through extensive cell death consistent with a function of *Pax2 *in stabilizing cell survival [3,8]. Our data confirm these earlier findings on our specific mutant background for the growing cochlear duct (Fig. 3E-H). However, while cell death as shown by pyknotic profiles was clearly associated with the cochlear duct, a large proportion of dying cells was in the mesenchyme around the growing cochlear duct (Fig. 3E-G). This distribution is suggestive of a non-cell autonomous survival role of *Pax *genes as suggested in the renal system. In addition to these cells, the forming spiral and vestibular neurons also showed massive cell death that was, however, less localized compared to the morphogenetic cell death in the cochlea (Fig. 3H). Consistent with a previous expression study reporting *Pax2 *expression in the medial wall of the otic capsule separating the forming cochlear duct from the brain, we found no evidence for the formation of cartilage in this area in *Pax2 *null embryos, therefore abutting the forming spiral and vestibular neurons and the cochlea next to the brain. In contrast to the absent medial cartilage, lateral cartilage was normal in *Pax2 *null inner ears (Fig. 3E).

**Figure 4:**
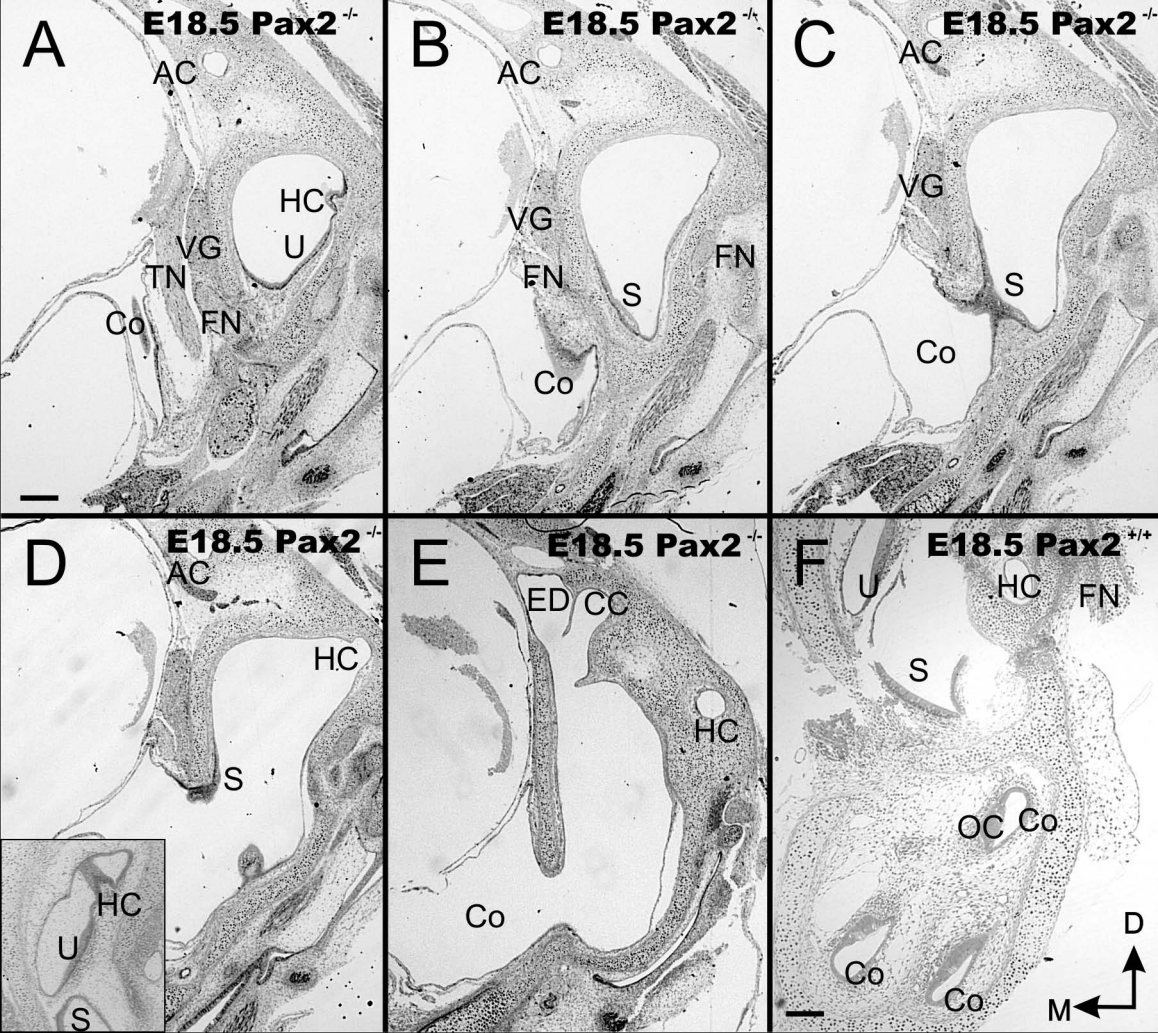
**Overview of sections through E18.5 ear**. These near serial coronal sections through the ear show the unusual morphology with a vesicle within the otic capsule (top of each section) and the large ventral vesicle that is expanded into the brain cavity, labeled as cochlea (C in A-E) instead of the multiple cross sections through the cochlear duct found in wild-type (F). Note the brain has been removed to verify before sectioning that a vesicle was present. Canals and canal cristae can be found along the superior vesicle surrounded by periodic mesenchyme. However, the horizontal crista is not in the horizontal canal (HC in A, D) whereas it sits in wild-type at the orifice of the horizontal canal (insert in D). Hair cells of the utircular and saccular macula are continuous and extend into the ventral, extruded sack of the ear (A-D). While adjacent to each other, the endolymphatic duct (ED) and the common crus (CC) are nevertheless distinct anatomical entities (E). AC, anterior canal/crista; CC, common crus; FN, facial nerve; ED, endolymphatic duct; HC, horizontal canal/crista; Co, cochlea duct/sack; OC, organ of Corti; PC, posterior canal/crista; S, saccule; TN, trigeminal nerve; U, utricle; VG, vestibular ganglion. Bar indicates 100 um.

These data are in close agreement with previous work, except that a cochlear duct apparently forms at later stages of development in *Pax2 *null mice. In fact, in all E18.5 *Pax2 *null mice examined (10 ears total), we found a tubular expansion without any separation of the superior part of the ear crowned by what appeared to be shortened semicircular canals (Fig. 3I-K). In addition we found an inferior part of the ear consisting of an enlarged sack instead of a coiled cochlea. This sack was extruded through a small foramen medial into the brain case (Fig. 4A). We also investigated whether this unusual and previously unnoticed morphology of the ear could be enhanced with *Pax8 *haploinsufficiency but found nearly identical ears in *Pax2 *null mice combined with *Pax8 *heterozygosity. In addition to the facial nerve passing around the constriction between the enlarged upper and lower half of the ear we found a sizable, albeit distorted vestibular ganglion (Fig. 3I-K).

We next wanted to understand the organization of these ears and conducted a detailed histological serial analysis (Fig. 4). Previous work on comparably old ears showed agenesis of the cochlea but near normal development of the vestibular region [5] whereas others found also vestibular expansion [3,4] or a prolapsed part of the ear in the brain case in some older mice [18]. Our histological data showed an expanded vestibular system with shortened canals and a continuous utricle, saccule, and cochlea (Fig. 4). The saccule continued into a sack-like expansion (Fig. 4C) of what should be a coiled cochlear duct (Fig. 4F). This sack or large vesicle extruded in almost all of our older samples from the otic capsule into the cranium (Fig. 4A-E). The saccule, identified based on its topology opposite to the stapes foot plate, continued across a foramen into the cochlear sack (Fig. 4C, D). All three sensory epithelia of the canal crista could be identified but only the anterior canal crista was near normal in organization and distribution (Additional file 1). The horizontal canal crista was found to be an isolated patch near but not adjacent to the horizontal canal opening (Fig. 4A).

Obviously, neither the utriculo-saccular foramen nor the ductus reunions form in the *Pax2 *null mice thus blocking the separation of utricle from saccule and cochlea (Fig. 4A,F). As will be apparent from the innervations described below, it is likely that the extruded sack represents the cochlear duct. However, there is no indication of the formation of an organ of Corti. A continuous patch of hair cells that extended from the saccule through the cochlea foramen and into the cochlea might be a combined saccule/cochlea (Additional File 2) or simply an expansion of saccular hair cells. In summary, while *Pax2 *null mice show clear evidence for formation of sensory epithelium, including hair cells immediately next to the opening, this epithelium clearly does not extent any further into the large vesicle in the brain leading to what appears essentially an empty vesicle lined by generalized otic epithelium.

### Nerve fibers reveal the presence of all sensory epithelia primordia during development

We next wanted to visualize the pattern of innervation as an indicator of the potential nature of the sensory epithelia. While the molecular details of their navigation across the ear to reach their specific sensory epithelia remains unclear, multiple sets of data show that growing nerve fibers can identify their target even if they are poorly segregated [19], do not develop sensory organs [20] or lack hair cell development [21].

**Figure 5:**
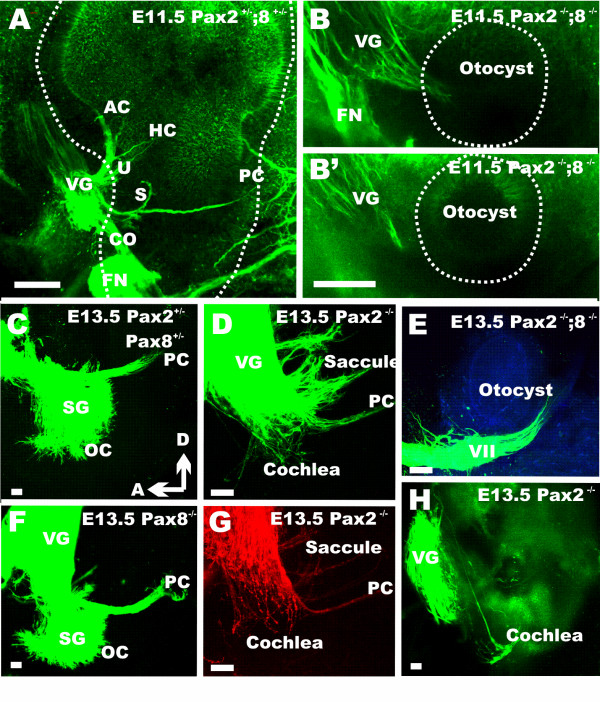
**The pattern of innervations at E11.5 and E13.5**. Innervation of the ear in terms of distinct fibers to sensory epithelia develops around E11.5 in wild-type mice (A). In *Pax2;8 *double null mutants there is at least a transient projection to a small otocyst which never develops any distinct projection pattern to the otocyst. At later stages, *Pax2;8 *double null mice have only an ear vesicle without any feature that is completely devoid of any afferent or efferent innervation (E, compare to C, D). In contrast, *Pax2; Pax8 *double heterozygote (C) and *Pax8 *null mouse (F) show at E13.5 fibers reaching to the posterior crista (PC) and the spiral ganglia (Sg) extend some fibers to the developing organ of Corti (OC). In contrast, fibers to the PC are reduced in *Pax2 *null mice, the saccular innervation is diffuse and there is little extension of afferent (D) or efferent (G) nerve fibers to the cochlea. Note the similarities in distribution of afferents (D) and efferents (G) in these two *Pax2 *null mice indicating a consistent but different pattern of innervation for these mutants. Injection of dye selectively into the otocyst reveals a few fibers extending toward the brain from the region of this presumptive cochlea (H). These data suggest that at least some early development of the cochlea neurons occurs but that the overall development or projection to the brain is severely truncated. OC, organ of Corti; PC, posterior crista; SG, spiral ganglion; VG, vestibular ganglion; VII, facial nerve, Bar indicates 100 um.

The earliest consistent fiber growth to the developing ear can be imaged at E11.5 [22,23]. At this stage fibers were already targeting the undeveloped regions of the future canal cristae, utricle and saccule in the wild-type (Fig. 5A). While a vestibular ganglion with fibers extending to the small otic vesicle formed in *Pax2;8 *double null mice (Fig. 5B.B') there was no indication of branching of these fibers at the otocyst. Consistent with previous work on *Eya1 *null mice [24], these data suggest that some neurons can at least transiently form in these mutants.

Using tracing with carbocyanine dyes [25] on the various mutant lines we found no defects in the *Pax8 *null mice (Fig. 5C,F) with a normal formation of spiral ganglia innervating the developing cochlea and other sensory epithelia like the saccule and posterior canal. In contrast, there was a massive defect in the *Pax2 *null mice which showed disorganized fibers projecting to the saccule and posterior canal and few dispersed spiral ganglion neurons (Fig. 5D). Injection of dye into the ear resulted in prominent labeling of vestibular neurons but also a few fibers emanating from the truncated ventral part of the ear that were suggestive of cochlear afferents (Fig. 5E). Efferent fibers could also be traced into the ear of *Pax2 *null mice and showed a pattern very similar to that of afferents (Fig. 5G).

As expected from the small vesicle we found in *Pax2;8 *double null mice at E10.5 and 11.5 (Figs. 2B, 3D, 5B) only a small vesicle was present at E13.5 (Fig. 5H). In contrast to the earlier stages where we detected some ganglion cells with fibers extending toward the ear (Fig. 5B), no nerve fibers were found extending to the small vesicle at E13.5 (Fig. 5H). Considering that *Pax2;8 *double mutant ears showed *Jag1 *and *Eya1 *expression in double mutant inner ears (Fig. 2), this suggests a rapid loss of sensory neurons likely by apoptosis as in *Pax2 *null mice (Fig. 3H). Combined these data suggest that in addition to promote otic vesicle formation, *Pax2 *and *Pax8 *combined are needed for neurosensory system differentiation and/or maintenance.

**Figure 6:**
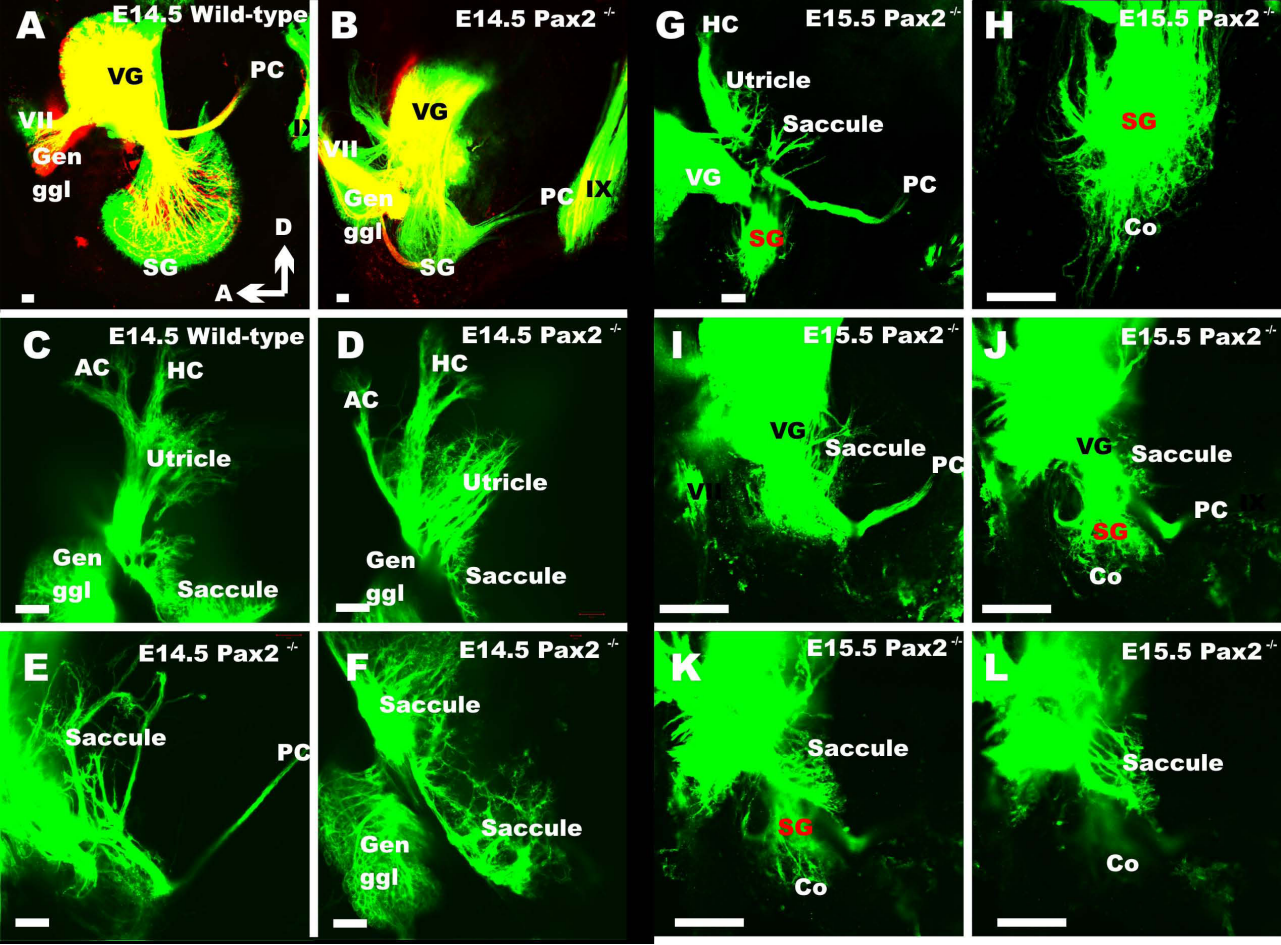
**The pattern of innervation at E14.5 and E15.5**. Wild-type animals show afferents (in green) to the growing cochlear spiral (A). Efferent fibers, shown in red, begin to form the intraganglionic spiral bundle. There is no cochlear spiral or spiral ganglion in *Pax2 *null mice (E). Instead there is an aggregation of neurons near the reduced fibers leading to the posterior canal crista (B), the presumed spiral ganglion (SG). In mutants, efferent fibers reroute from the spiral ganglion to the facial nerve (B). The innervation to the anterior canal crista (AC in C, D) is reduced, but not that to the horizontal crista (C, D). The saccule shows near normal organization of fibers in the sensory epithelium (F) as well as an overshooting projection at a different focal plane (E). At E15.5, sensory neurons that can be filled with brain injections of lipophilic dyes form a tear drop arrangement of spiral sensory neurons (G, H). Multiple nerve fibers radiate away for a short distance toward the cochlear expansion (CO), but no pattern reminiscent of radial fibers in the cochlea can be observed (G, H, J, K). There is a variable reduction of nerve fibers to the anterior and posterior canal crista (D, G) and the innervation of the saccule is variable (G-L). A focal series (I-L) shows that spiral ganglion neurons are distinct in their distribution from vestibular ganglion cells as is evidenced by. AC, anterior canal crista; HC, horizontal canal crista; Co, cochlear duct/sack; Gen Ggl, geniculate ganglion; PC, posterior canal crista; VG, vestibular ganglion; IX, glossopharyngeal nerve. Bar indicates 100 um.

We next investigated the innervation of the ear in later stage *Pax2 *mutant embryos. Starting at E14.5 (Fig. 6A,B) we found prominent differences in the development of the cochlea spiral ganglion. In wild-type the spiral ganglion formed an elongated extension curved along the cochlear duct whereas the spiral ganglion formed a tear drop addition to the vestibular ganglion with little longitudinal extension. The difference in topology was particularly obvious when comparing the topology of nerve fibers projecting to the posterior canal. These were reduced in *Pax2 *null mice (Fig. 6B) and emanated from vestibular neurons mixed to what might be part of the spiral ganglion. In contrast to these massive differences, the innervation to the anterior canal crista and utricle were only different in terms of size compared to wild-type littermates (Fig. 6C,D). The saccule consisted of two small patches of innervation but also numerous fibers radiating around the medial wall of the otocyst, including a few fibers extending toward the posterior canal crista. Even compared to the younger wild-type ears (Fig. 5) this fiber tract was reduced. This data suggests that spiral neurons form but never segregate from the inferior vestibular ganglion.

**Figure 7:**
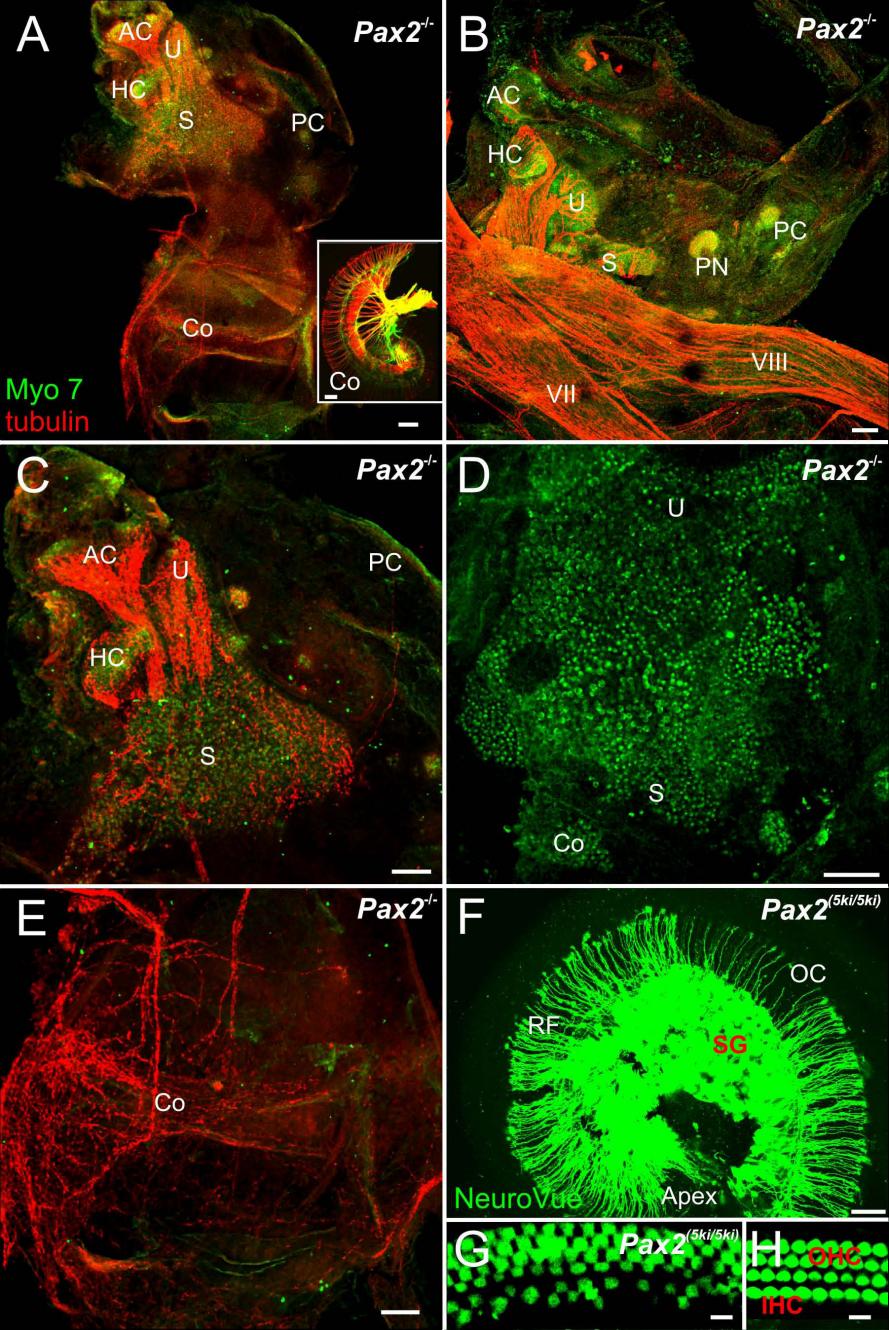
**Vestibular hair cells and some innervation develop in *Pax2 *null mice**. Pattern of innervations and distribution of hair cells as revealed by anti-tubulin (red) and anti-Myo 7 (green) antibodies (A-E, G, H) and lipophilic dye tracing (insert in A, F). Hair cells are concentrated to the superior part of the ear in *Pax2 *null mice (A, C, D), innervations exists to vestibular (A-C) and cochlear sack (A, E). *Pax2 *^*5ki/5ki *^show a near normal innervation of the cochlea (F) compared to that found in wild-type (insert in A). Anterior, horizontal and posterior canal and crista can be recognized in all ears (compare C, F) and may receive no or few nerve fibers. Myosin 7 labeled hair cells form an unstructured aggregation tentatively divided into utricle (U) saccule (S). In addition to several small isolated patches of hair cells we found aeas without hair cells near the ventral border of the saccular patch (A-D). Innervation implies that nerve fiber pathfinding is unaltered. The cochlear sack has no hair cells and a diffuse innervation. This contrasts to the radial fibers (RF) to the inner and outer hair cells (IHC, OHC) of the organ of Corti (OC) with the in *Pax2 *^*5ki/5ki *^(F, G) and wild-type cochleae (insert in A, H). AC, anterior canal crista; HC, horizontal canal crista; IHC, inner hair cell; OC, organ of Corti; OHC, outer hair cell; PC, posterior canal crista; RF, radial fibers; S, saccule; SG, spiral ganglion; U, utricle. Bar indicates 100 um.

At an even later stage (E15.5) we found many fibers radiating from the tear-drop shaped spiral ganglion toward the cochlear sack (Fig. 6G,H). Investigating various focal planes following dye injection (Fig. 7I-L), we found that spiral ganglia were clearly distinct from inferior vestibular ganglia; the latter projected to the saccule and the posterior canal at this stage. As in younger stages, the fibers to the posterior canal were disorganized and few. Together these data show that *Pax2 *null mutant embryos can form some cochlear sensory neurons which extend projections to the brain. However, these never develop to a typical spiral ganglion.

### Utricular, saccular and cochlear hair cells are continuous

**Figure 8:**
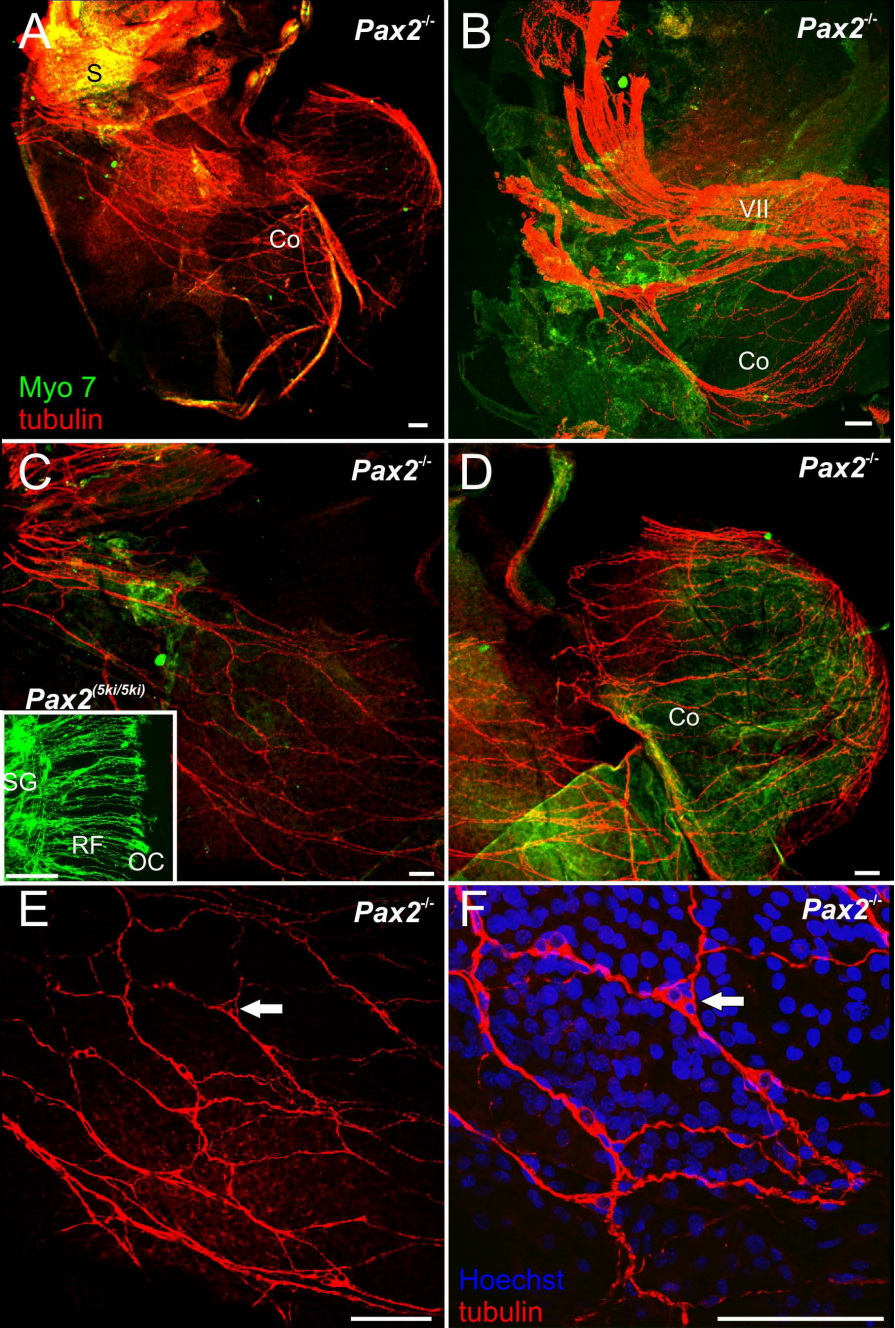
**A cochlear sack innervation develops as dispersed neurons in *Pax2 *null mice**. Details of the innervation of the cochlear sack are shown using anti-tubulin immoncytochemistry in *Pax2 *null mice (red, A-F) and lipophilic tracing in wild-type mice (insert in C). In contrast to the patterned innervation of the organ of Corti in wild-type mice by radial fibers emanating from spiral ganglia (insert in C) innervation of the cochlear sack of the mutant is provided by an irregular network of nerve fibers (A, C, D). A continuity of this network with the vestibular fibers (B) could not be verified by tracing from the brain, suggesting that even the fibers that extent toward the saccule may not project to the brain for labeling. Higher magnification (E, F) shows a network of neurons that appears to be interconnected by fibers thus generating the network that is so prominent at lower magnification throughout the cochlear sack. Bar indicates 100 um.

We next investigated at E18.5 the pattern of innervation and the distribution of nerve fibers using anti-tubulin and anti-Myo VII to highlight nerve fibers and hair cells, respectively. As was the case with the lipophilic dye tracing experiments, the pattern of innervation of the anterior and horizontal canal crista and utricle was fairly normal in *Pax2 *mutant embryos (Fig. 7A-C). However, there was no clear pattern of innervation suggestive of distinct boundaries of the saccule and the fibers to the posterior canal crista were reduced or entirely absent. After physically removing the facial nerve and the vestibular ganglion, an unusual and occasionally dense innervation of the ventral cochlear expansion was found (Fig. 7E). These fibers concentrated on the anterior aspect of the large ventral sack without any clear boundary recognition. Comparing several ears showed that fibers were always present but their distribution varied dramatically (Fig. 7,8).

Hair cells were identified using Myosin VII immunocytochemistry and found in all three canal cristae, but the shape of the cristae and their size deviated variably from normal. Hair cells of the utricle and saccule were contiguous and extended across the constriction separating the superior from the inferior part of the ear (Fig. 7C,D). Assuming that only hair cells inferior to the constriction are cochlear hair cells, a cochlear duct, some cochlear duct innervation, a spiral ganglion but apparently no cochlear hair cells form in these ears. This suggests that the cochlea duct forms, but with a delay and in an unusual shape including the separation of all elements and a complete absence of any organ of Corti like histogenesis of hair cells.

At this stage we also studied the *Pax2 *^*(5ki/5ki) *^genotype (Fig. 7F) as this allelic combination acts as a hypomorph in the embryos due to a gene dosage level between *Pax2*^*-/- *^and *Pax2*^*+/- *^[17]. While the overall organization and innervation of *Pax2 *^*(5ki/5ki) *^ears was indistinguishable from normal mice, the detailed patterning of hair cells in the cochlea showed minor aberrations such as multiple and somewhat disorganized rows of hair cells (Fig. 7G,H) indicative of a role for *Pax2 *and *Pax5 *in hair cell patterning as also noticed in zebrafish [8].

An interesting aspect of the *Pax2 *mutant ear phenotype was the dense innervations of the cochlear sack (Fig. 8), which showed a meshwork of criss-crossing fibers instead of radial bundles emanating from the spiral ganglion to reach the organ of Corti. This meshwork of what appeared to be nerve fibers spinning around the cochlear sack proved upon closer inspection to be a network of fibers emanating from small, MYO7 positive cells, which likely are neurons embedded into the wall of the cochlear sack (Fig. 8C-F). These data suggest that cochlear sensory neuron formation may occur but that there is no migration of those neurons away from the cochlear sack and they do not develop any clear pattern of innervation reminiscent of the radial fibers in normal innervation (Fig. 8C and insert). It is not clear what neurotrophic support maintains those neurons as neither supporting cells nor hair cells are found in the area of the cochlear sack supplied by these nerve fibers. Clearly, there is cooperation between Pax proteins and even functional equivalence as revealed by the *Pax2 *^*5ki/5ki *^data. Recent work in flies has suggested that *dPax2 *has a very complicated promoter regulation [26] and it is likely that similar complexity might play a role for the differential expression regulation in mouse (and mammalian) *Pax2;5;8 *expression.

## Discussion

Previous work on several *Pax2 *mutant mice has suggested that *Pax2 *affects the vestibular system to a variable degree [3-5]. We provide here the first detailed histological and whole mount analysis of the *Pax2 *null phenotype, which revealed new unappreciated features. Our data suggest a Mondini type dysplasia of the cochlea and vestibular system with short canals and a shortened endolymphatic sack and common crus, much like in *Foxi1 *null mice [27]. In this paper we analyzed for the first time the innervation defects in *Pax2 *null, *Pax2;Pax8 *double null mice and show that innervation of the *Pax2 *null mouse ear has a delayed and truncated formation of spiral ganglia. As previously reported [4], cell death occurs in both the sensory neurons and the cochlear duct [3] but initial formation of sensory neurons is near normal as suggested previously [3]. However, the *Pax2 *phenotype described thus far has been overstating the degree of loss of the cochlea which in fact forms as a featureless sack that extrudes in the brain case lumen in older mouse embryos.

### Effects of *Pax2; Pax8 *double null on ear development

We show for the first time the phenotype of the *Pax2; Pax8 *double null mutants. These data show that *Pax2; Pax8 *mutant otic vesicles are much smaller than their normal counterpart possibly because the *Pax2; Pax8 *double null ear has an incomplete invagination with extensive cell death of non-invaginated tissue. This interpretation is consistent with recent findings in chicken that suggest that Pax2 affects cell shape and ear morphogenesis [13]. Surprisingly, even these reduced vesicles show in mice several transcription factors almost normally expressed, and develop initially some neurons but seems to be unable to sustain neurosensory development. *Pax2;8 *mutants are in that respect reminiscent of *Eya1 *null mice which show also a transient formation of neurons but no indication of sensory differentiation [24].

A limited development of only a vesicle with no evidence for sustained neurosensory formation as we describe here in *Pax2;8 *double null (Fig. 1,2,4) has thus far been reported for a small number of ear mutants such as the *Fgf3;10 *double null mice [28,29], the *Gata3 *null mice [30] and the *Eya1 *null mice [18]. Previous work has established that *Fgf3 *and *Fgf10 *together are needed to develop an ear past the placode and otocyst stage [28,29] possibly by guiding neuronal differentiation of the otic ectoderm through suppression of BMP signaling [16,31]. Consistent with this interpretation is that *Pax2;8 *are regulated by *Fgf's *and appear to be downstream to the *Fgf *activation [32]. Indeed, some reduction of *Pax2 *expression has been noted in *Fgf3;10 *null ears [28]. The data on *Pax2;8 *double null mice clearly show that the ear is truncated in development at the level of the otocyst (Fig. 1,2,4). *Pax2;8 *double mutants are delayed in otic placode invagination, and the remaining otocyst shows near normal expression of some genes, suggesting that the otocyst is still responsive to ventralizing signals such as sonic hedgehog [33]. Yet, neuronal development is only transient and could not proceed normally beyond E11.5 (Fig. 5). Further work will need to demonstrated the overall reduction in crucial genes for sensory development such as *Neurog1 *[34,35] and *Neurod1 *[36,37] in such *Pax2;8 *double null mice.

Previous work [18] has compared the early embryonic expression pattern of *Eya1*, *Pax2 *and *Pax8 *using *in situ *hybridization and investigated the ear in *Pax2*, *Eya1 *and *Six1 *compound mutants with latex paintfilling. These data suggest that *Pax2 *interacts with *Eya1 *during inner ear morphogenesis, in particular in sensory areas of the inner ear. Our data, however, indicate that *Eya1 *is not a genetic target of *Pax2;8 *in this system as *Pax2;8 *does not regulate the expression of *Eya1 *(Fig. 2) and some *Pax2 *expression persists in *Eya1 *null mice [38]. In renal development, Pax2 and Eya1 proteins are known to complex on the Gdnf promoter [39] and Gdnf is also expressed in the ear [40,41]. Further work is needed to show the remaining expression of *Gdnf *in *Pax2;Eya1 *compound mice. *Pax2;8 *combined are needed for sustained neurosensory ear development and normal inner ear morphogenesis. *Pax2 *and *Pax5 *maintain hair cells and direct nerve fiber growth in zebrafish [8] and at least *Pax2 *seems to play a similar role for the cochlea of mice. Hence, as previously suggested based on expression of a *Pax2;6 *fusion protein in 'eyes' and 'ears' of the boxed jelly fish [2] as well as other expression similarities in bHLH and other genes [42], these data clearly establish that *Pax *gene expression is essential for normal neurosensory development of eyes and ears, two major sensory systems of the vertebrate head.

### *Pax8 *alone has no defects on embryonic ear development

*Pax8 *is one of the first genes expressed in the developing mouse ear [7,10,43] but this early placodal expression is rapidly replaced by *Pax2 *in ear development. Lineage tracing experiments previously showed that the entire inner ear is derived from *Pax8*-positive cells [44]. In spite of this, we could not detect any defects in sensorineural development in the absence of *Pax8 *(Fig. 3,5) suggesting that the early expression of *Pax8 *can be functionally compensated by *Pax2 *expression. However, previous work [45] has shown that *Pax8 *null mice are profoundly deaf due to athyriodism [11] because of maturation defects of the ribbon synapses connecting hair cells to afferents [46,47]. This defect can be nearly fully compensated for by postnatal thyroid T(4) treatment [45], preferentially already in late embryos to ensure normal hair cell maturation and synaptogenesis.

As is apparent through the data of the *Pax2;Pax8 *double null and the clear defects known in *Pax2 *null mice, *Pax8 *cannot compensate for *Pax2 *in late sensorineural development but is necessary in combination with *Pax2 *for early ear development. What exactly the function of *Pax8 *is in ear development and how the absence of both cause the incomplete invagination and at the most transient formation of some neurons remains to be demonstrated. In analogy with other systems [48] we assume that *Pax8 *simply cannot compensate for *Pax2 *because it is not expressed any more in the ear when *Pax2 *is needed, in particular in the cochlea [18]. Testing this assumption would require to replace *Pax2 *by *Pax8 *which likely will result in normal ear development as already shown for *Pax5 *replacement of *Pax2 *[17] and verified here (Fig. 8,9) As with the *Pax2 *^*5ki/5ki *^mice, there may be subtle differences like alignment of hair cells that would allow to elucidate further the differential function, if any, of *Pax2 *and *Pax8 *in ear development that is independent of the expression difference. However, it is very likely that the regulatory differences in expression through the complex promoter system recently described in *dPax2 *[26] rather than the minor sequence differences in protein will determine the function of *Pax *genes.

### *Pax2 *has defects in vestibular and cochlear development

#### Vestibular defects

Previous work has identified a variable phenotype in the vestibular system of *Pax2 *null mice [4,5] that was later related to indirect effects as most vestibular development appeared to progress normally [3]. Our data on early development is in line with these considerations. However, there are clear defects in later development that cannot be related to a near normal vestibular system development. Most notably are the disorganization of the canal cristae and alterations in the relative position of the horizontal canal relative to the horizontal canal crista. The latter may form in the lateral wall of the superior part of the bipartite otic vesicle several hundred microns away from the horizontal canal. In agreement with two of the previous three papers those alterations are comparatively minor and require serial sectioning to be detected and thus could have been missed easily. In contrast to claims raised based on paint fillings suggesting a fusion of the common crus with the endolymphatic duct [3], our data show that both structures form as distinct albeit closely associated entities that most likely could not be discriminated at the lower resolution of a whole mounted paint filled ear (Fig. 4). In summary, the overall morphological changes in the superior part of the bipartite otic vesicle are mild and it remains unclear how direct or indirect they relate to the lack of *Pax2*.

A major alteration is the organization and separation of the utricle, saccule and possibly cochlea sensory epithelium. Data on early gene expression show that *Pax2 *expression overlaps with the *Lfng *expression as early as E10.5 and thus could affect at least the utricular and saccular maculae [3]. Indeed, some defects were noted previously in the saccule but since this study terminated at E15.5 it could not fully evaluate the effects of *Pax2 *on hair cell distribution and differentiation. We show here an expansion and fusion of hair cell patches that, judging from their pattern of innervations, belong to the utricle, saccule and possibly cochlea (Fig. 7). Major effects on hair cell formation were noted previously in zebrafish but none have been reported in the three previous analysis of ear development in *Pax2 *null mice likely either because no specific hair cell markers were used [4,5] or the analysis was not extended into older embryos were this defect is so obvious [3]. Indeed, recent data suggest a long term expression of *Pax2 *in post-hatchling chicken in differentiated hair cells [49] and *Pax2 *and *Pax5 *are important for normal hair cell development in zebrafish [8] and *Pax2 *is later expressed in hair cells [3,50].

Current data suggest that non-sensory epithelium specification is as tightly regulated as sensory epithelium specification and this specification is needed for segregation of sensory epithelia and their normal development requiring a multitude of genes to achieve this segregation [51-53]. As was recently shown in *Lmx1a *null mice [19,54], expression outside the sensory epithelia can cause major defects in overall ear development and segregation of sensory epithelium. Clearly, the maculae of the utricle and saccule are not segregated in *Pax2 *null mice implying that *Pax2 *interacts with other genes thus far unidentified in this sensory segregation through the specification of non-sensory domains in the ear.

#### Cochlear defects

Our data agree with previous work that cochlear outgrowth is delayed and this delay correlates with excessive cell death [3] in the immature cochlear duct (Fig. 3). As previously noted [4,5,18] there is the possibility of a late formation of a cochlear sack instead of a cochlear duct. We show here that this is actually a fairly regular feature of late development and in most instances leads to the prolaps of this sack into the brain case, extending both 'cochlear sacks' underneath the brainstem to meet near the midline. This prolaps seems to happen at areas of excessive cell death in the mesenchyme around the growing cochlear duct and may relate to the absence of cartilage formation medial to the cochlea that normally separates the ear from the brain case. This expanded ventral 'cochlear sack' shows little differentiation in *Pax2 *null mice with sensory epithelium forming only adjacent to the constriction through which it expands (Figs. 3,4,7,8). This sensory epithelium is in continuation with the saccule and it remains unclear if this is a non-segregated part of the cochlea or a part of the saccule. Likewise, normal ear development requires formation of the utriculo-saccular foramen to segregate the utricle form the saccule [52,55] and the formation of the ductus reunions that constricts the ear between the saccule and the basal turn of the cochlea. Molecularly, the utriculo-saccular foramen depends in part on *Otx1 *[53,56] whereas the ductus reunions depends on *Lmx1a *[19].

### Innervation defects

Previous work has demonstrated very early overlap of *Eya1 *and *Pax2 *in early ear development [18] whereas later stages show no expression of *Pax2 *in sensory neurons but in hair cells [49,50]. *Pax8 *and *Pax2 *reporter expression show extensive expression in all sensory neurons as well as sensory epithelia [57,58] suggesting that at least at some time during development there is expression of either of these *Pax *genes in neurosensory precursors. Our data show little defects in the innervations of several vestibular epithelia of *Pax2 *mutant ears, except for the saccule and posterior canal (Fig. 5, 6,7,8). Whether these defects are a consequence of the patchy absence of hair cells in the confluent utriculo-saccular epithelium or reflect a primary defect in these vestibular sensory epithelia remains unclear. Disorganized innervations of the saccule could relate to alterations in numerous genes now identified in neuronal differentiation, in particular *Neurod1 *[37].

Most interesting is the pattern of innervation of the 'cochlear sack' and the distribution of sensory neurons associated with this innervation. There was a clear indication of spiral ganglion formation near the inferior vestibular ganglion innervating the saccule and posterior canal which radiated a few fibers to the cochlear sack (Figs. 5,6). In contrast to this tracing data, later examination of the innervations using tubulin as a neuronal marker showed a dense meshwork of innervations in particular of the medial part of the 'cochlear sack' (Figs. 7, 8), suggesting that the distributed sensory neurons may not project to the brain and can thus not be filled by dye tracing (Figs. 5, 6). Interestingly these fibers emanated from neurons embedded singly or in clusters in this network (Fig. 8) suggesting that sensory neurons form and survive. At E18.5 sensory neurons that are not in contact with neurotrophin releasing sensory epithelia are either dead or dying [59]. How these neurons survive beyond the normal loss induced by the absence of neurotrophins emitted from sensory epithelia remains unclear. However, given that *Ntf3 *is the main supporting neurotrophin for spiral neurons [48,60] and is expressed in supporting cells it may be possible that a certain level of differentiation of the organ of Corti supporting cells takes place in the absence of any hair cells and that this differentiation is sufficient to express enough *Ntf3 *to support neuronal survival. Similar data are known for *Atoh1 *null mice in which a targeted innervation of the cochlea develops in the absence of hair cell differentiation and enough neurotrophin is expressed to maintain innervation at least until birth [21].

This diffuse formation of what appears to be spiral sensory neurons distributed within the wall of the 'cochlear sack' needs to be further investigated in terms of their birthdate using BrdU [61] and survival through neurotrophic factors.

## Conclusion

Our data demonstrate that all three *Pax2;5;8 *genes can signal redundantly in ear development with their respective function depending on spatio-temporal expression pattern. *Pax2 *can compensate for *Pax8*, but loss of both results in otocyst formation with only transient expression of sensory markers and development of some neurons. Likewise, a *Pax5 *minigene knocked into *Pax2 *can nearly completely compensate for *Pax2*. We also show that *Pax2 *null does not affect the early neurogenesis, but results in the formation of a cochlear sack with rather aberrant innervation patterns later in development.

## Methods

### Mice

The *Pax2 *and *Pax8 *null mice have been previously described [14,17] and these lines were bred to homozygosity as single or double knockout mice generating the expected frequency of mutant embryos in Mendelian ratios. *Pax2;8 *compound female carriers have defects in their genital tracks and only a small portion are fertile thus leaving us with a very small number of *Pax2;8 *double null mutants to work with. The *Pax2*^*5ki/5ki *^mice, in which the *Pax2 *gene was replaced by a *Pax5 *minigene, were also previously described and bred according to those description [17].

Embryos were collected from timed pregnant females at E8, 9.5, 10.5, 11.5, E13.5, E14.5, E15.5 and E18.5 counting noon of the day the vaginal plug was found as E0.5. Pregnant mothers were anesthetized with a lethal dose of Avertin (1.25% of 2.2.2-tribromoethanol at a dose of 0.025 ml/g of body weight). Embryos were perfused with 4% paraformaldehyde (PFA) in 0.1 M phosphate buffer (pH 7.4). Heads were isolated and fixed in 4% PFA for further analysis. Offspring was genotyped by PCR analysis of tail DNA as previously described [17]. All animal procedures were approved by the University of Iowa animal care committee according to IACUC guidelines for use of laboratory animals in biological research (ACURF #0804066).

### X-gal staining

Heads of mice perfused with 4% PFA were hemisected. Ears were dissected and then briefly washed with 0.1 M phosphate buffer. The samples were stained in a solution containing 0.1 M phosphate buffer, 0.01% deoxycholic acid, 0.02% NP40, 2 mM magnesium chloride, 5 mM potassium ferricyanide, 5 mM potassium ferrocyanide and 0.1 mg/ml X-gal (5-bromo-4-chloro-3-indolyl-β-D-galactoside) for about 24 hours at room temperature [62].

### *In situ *hybridization

In situ hybridization was performed as described [63] using the RNA probe labeled with digoxigenin. The cover slipped slides were viewed in a Nikon Eclipse 800 microscope and images were captured with Image-Pro software.

### Immunofluorescence

For immunofluorescence staining, the ears were dehydrated in 100% ethanol overnight and rehydrated and blocked with 0.25% normal goat serum in PBS containing 0.01% Triton-X-100 for 1 hour. Then the primary antibodies for Tubulin (Cell Signaling Technology) and Myo VII (Myosin VIIa, Proteus Biosciences) were used in dilutions of 1:800 and 1:200 respectively, and incubated for 48 hours at 4°C. After several washes with PBS, corresponding secondary antibodies (1:500) (Alexa fluor molecular probe 647 or 532; Invitrogen) were added and incubated overnight at 4°C. The ears were washed with PBS and mounted in glycerol and images were taken with a Leica TCS SP5 confocal microscope.

### Lipophilic dye tracing

The heads of the mice were cut sagittally along the midline and different colored NeuroVue dyes were inserted to label the afferent and efferent fibers from and to the inner ear. Lipophilic dye-soaked filter strips [25] were inserted into the alar plate of the brainstem to label the eighth cranial nerve afferent fibers. Efferent fibers were labeled by applying dye into the olivo-cochlear efferent bundle as it crosses the floor plate in rhombomere 4. Half heads were kept in 60°C oven for about 3-7 days depending on the age of the mice for proper diffusion. In the E14.5 mice the dyes were injected in rhombomere 2, 5 and 7 to label the afferent and efferent fibers. Then the ears, vestibular ganglia and brains were dissected out for analysis and images were taken by Leica TCS SP5 confocal microscope.

## Authors' contributions

All investigators planned the experiments, MBo bred and collected the embryos, BF did the tract tracing and imaging, DdC did the histology and figure assembly; these three authors shared the majority of the writing, PX and MBu read and commented on the manuscript. All authors read and approved the final version.

## Supplementary Material

Additional file 1 **Detail of cochlear and saccule**. The higher magnified images show the formation of sensory epithelia with hair cells through (C) and on either side of the closed foramen though which the prolapsed cochlear sack extrudes. Co, cochlear sack; S, saccule. Bar indicates 100 um. Click here for file

Additional file 2 **Detail of canal organization**. All canal cristae can be identified, but the posterior canal crista is drastically reduced (A). Note that the horizontal crista is, like the anterior crista, very close to the utricle on the lateral wall of the utricular recess. However, this crista is not close to the horizontal canal opening. AC, anterior canal crista; HC, horizontal canal cirista; PC, posterior canal crista; U, utricle. Bar indicates 100 um. Click here for file
