# Supplementary figures and images for: Pax2 and Pax8 cooperate in mouse inner ear morphogenesis and innervation
Source: BMC Dev Biol. 2010 Aug 20;10:89. doi: 10.1186/1471-213X-10-89 (PMC2939565; doi:10.1186/1471-213X-10-89)

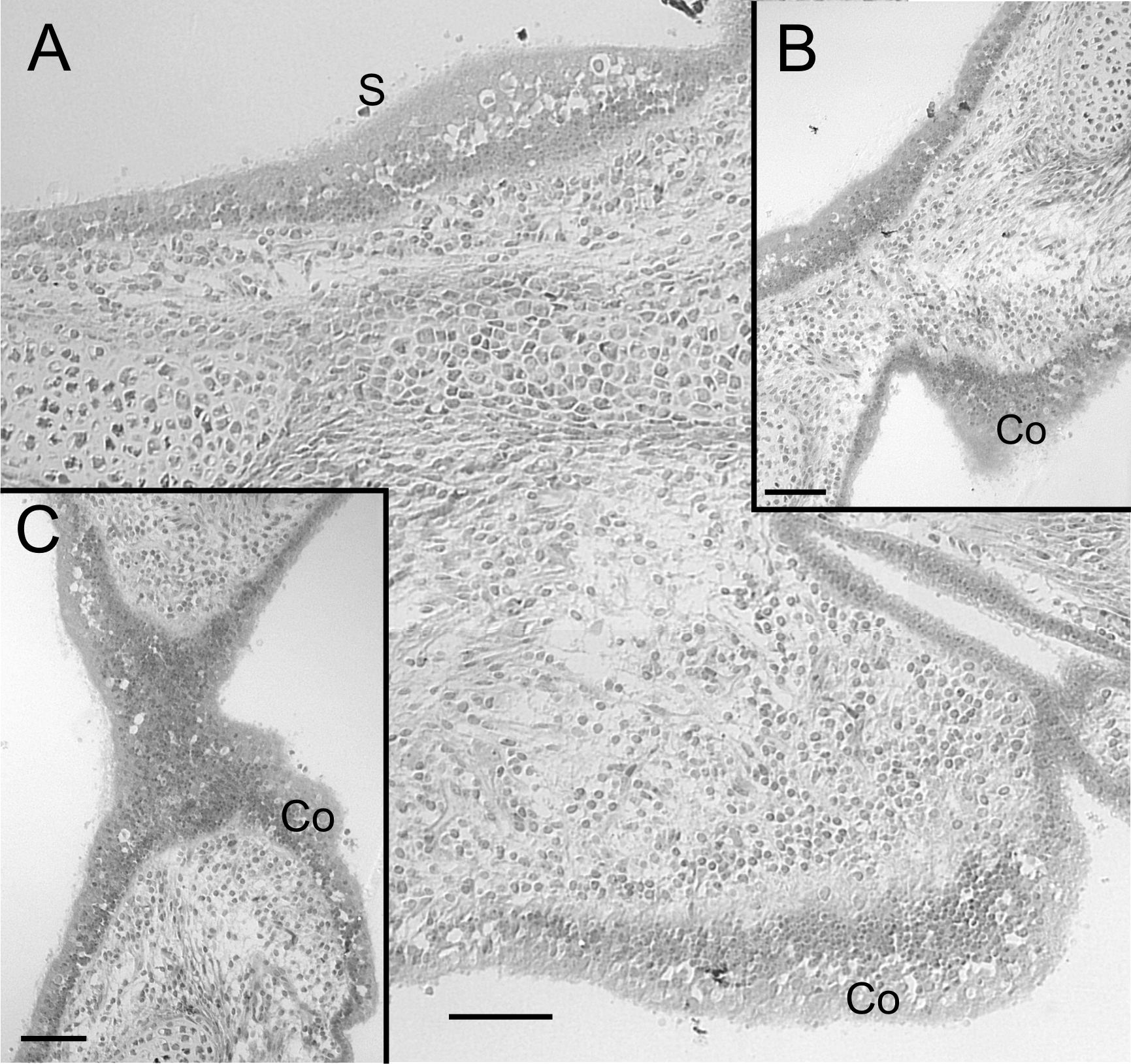

Supplement: Additional file 1 — Detail of cochlear and saccule. The higher magnified images show the formation of sensory epithelia with hair cells through (C) and on either side of the closed foramen though which the prolapsed cochlear sack extrudes. Co, cochlear sack; S, saccule. Bar indicates 100 um. [file 1471-213X-10-89-S1.TIFF]

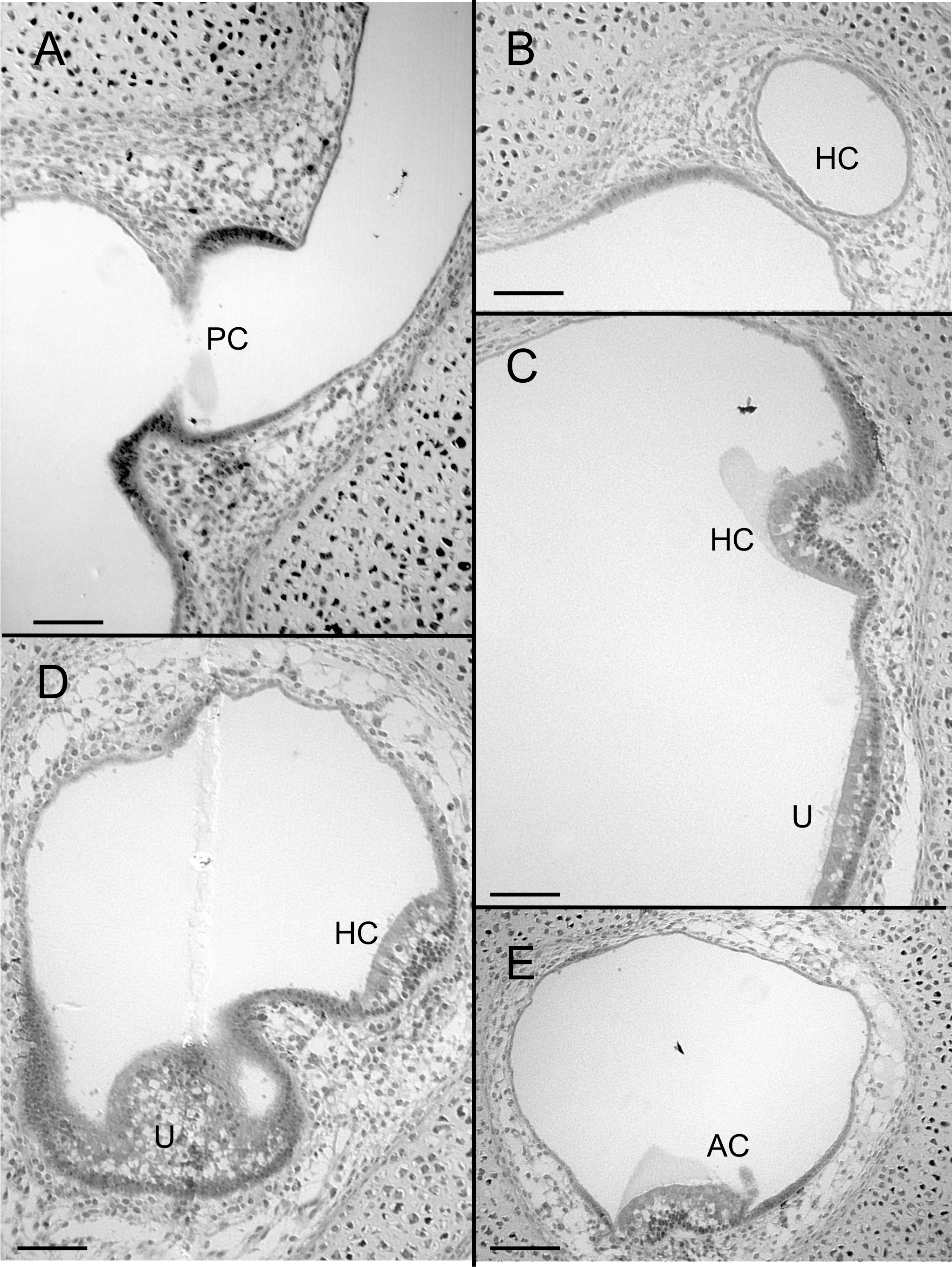

Supplement: Additional file 2 — Detail of canal organization. All canal cristae can be identified, but the posterior canal crista is drastically reduced (A). Note that the horizontal crista is, like the anterior crista, very close to the utricle on the lateral wall of the utricular recess. However, this crista is not close to the horizontal canal opening. AC, anterior canal crista; HC, horizontal canal cirista; PC, posterior canal crista; U, utricle. Bar indicates 100 um. [file 1471-213X-10-89-S2.TIFF]
